# Time-use movement behaviors are associated with scores of depression/anxiety among adolescents: A compositional data analysis

**DOI:** 10.1371/journal.pone.0279401

**Published:** 2022-12-30

**Authors:** Fernanda Rocha de Faria, Djalma Barbosa, Cheryl Anne Howe, Karina Lúcia Ribeiro Canabrava, Jeffer Eidi Sasaki, Paulo Roberto dos Santos Amorim

**Affiliations:** 1 Federal Institute of Education, Science and Technology of Triângulo Mineiro, Ituiutaba Campus, Ituiutaba, Minas Gerais, Brazil; 2 Department of Applied Social Sciences, Federal University of Rondonópolis, Rondonópolis, Mato Grosso, Brazil; 3 School of Applied Health Sciences and Wellness, Ohio University, Athens, Ohio, United States of America; 4 Federal Center for Technological Education of Minas Gerais, Contagem, Minas Gerais, Brazil; 5 Department of Sports Science, Federal University of Triângulo Mineiro, Uberaba, Minas Gerais, Brazil; 6 Department of Physical Education; Federal University of Viçosa, Viçosa, Minas Gerais, Brazil; Indiana University, UNITED STATES

## Abstract

Movement behaviors have been associated with mental health. The purposes of this study were to examine the association between movement behaviors and scores of depression/anxiety among adolescents and to determine the difference in depression/anxiety associated with reallocating time between different movement behaviors. This cross-sectional study included 217 Brazilian adolescents (15 to 18 years old, 49.3% female). Adolescents wore an accelerometer for one week to assess the four-movement behaviors which include sleep, sedentary behavior (SB), light physical activity (LPA), and moderate-to-vigorous physical activity (MVPA). The depression/anxiety score was calculated by factor analysis using the 12-item General Health Questionnaire. Compositional data analyses were used to examine the association between movement behavior and the depression/anxiety score. Compositional isotemporal substitution models estimated the change in depression/anxiety score associated with reallocating 10, 30, and 60 min between movement behaviors. The composition of movement behaviors was significantly associated with depression/anxiety scores (*p* < 0.05). Replacing time from SB to LPA was associated with improvement in the depression/anxiety score, while the inverse was associated with an increase in this score. Replacing time of LPA with MVPA was associated with worsening in the depression/anxiety score. The 24-h time distribution of the day may play a crucial role in mental health. Compositions with more time spent in LPA at the expense of less SB are associated with improvement in the scores of depression/anxiety. The type of MVPA may moderate its effects on depression/anxiety in adolescents. Holistic interventions including the full range of movement behaviors may be a gateway to reduce the levels of depression/anxiety in adolescence.

## Introduction

Sedentary behavior (SB) and physical activity have been associated with mental health. In fact, Choi et al. [1] by using a bidirectional Mendelian randomization approach in adults showed that physical activity, measured by accelerometry, is a protective factor against depression, presenting no reverse causality. Among adolescents, previous studies have shown a positive association between SB and depression/anxiety levels [2, 3], while moderate-to-vigorous physical activity (MVPA) appears to be negatively associated with it [4].

The finite 24-h period of the day can only be composed of a limited number of movement behaviors that include SB, MVPA, light physical activity (LPA), and sleep [5–9]. In this way, time spent in one movement behavior is intrinsically co-dependent of time spent on, at least, another movement [5, 10, 11]. That is, only one movement behavior can be done at a given time, so changes in time spent in one movement behavior (e.g., MVPA) can only occur by changing one or more of the other behaviors [5, 10, 11].

These full range of possible movement behaviors are compositional data in nature once the fixed amount of time of the day (24-h) establishes a perfect multi-collinearity among them [6, 7]. Due to this, recently, compositional data analysis has been applied in a few studies to evaluate the association between this type of data and health parameters [5, 7, 8, 10, 11]. This statistical technic expresses movement behaviors relative to the others through isometric log-ratio (ilr) coordinates [12], so that they can be interpreted together as co-dependents. Therefore, the use of compositional data analysis can provide a different insight into the data by highlighting the crucial role of the 24-hour daily time distribution among different movement behaviors on health parameters.

To date, just a limited number of studies have applied this statistical approach among adolescents [13–15]. These previous studies have involved a range of health indicators, such as body mass index (BMI), cardiometabolic biomarkers, and blood pressure [13–15]. However, to our knowledge, no study has applied compositional data analysis to evaluate the association between time spent in movement behaviors and signs of depression/anxiety in adolescents. In fact, just one study, conducted by Cruz et al. [16] among adults evaluated depression symptoms through this statistical method. Their results confirmed the positive association between SB and depression symptoms and pointed out that promoting sleep and MVPA may be helpful to reduce these symptoms [16].

Understanding the association between time spent in movement behaviors and depression/anxiety can help to tailor more integrative and effective interventions that address all movement behaviors to prevent and/or reduce mental impairment among this population. This study, therefore, aimed to examine: 1) the associations between time spent in the four possible movement behaviors (sleep, SB, LPA, and MVPA) within the finite time of a 24-h day and scores of depression/anxiety; and 2) how reallocations of time between these movement behaviors were associated with the depression/anxiety scores.

## Materials and methods

### Study design and participants

This was a cross-sectional study, carried out between March and September 2018 with a random and representative sample of adolescents enrolled in the technical high school of the Federal Institute of Education, Science, and Technology of Triângulo Mineiro, Ituiutaba Campus, Minas Gerais, Brazil. The study protocol was conducted according to the Declaration of Helsinki and was approved by the Human Research Ethics Committee of the Federal University of Viçosa, under the decision number 74104217.3.0000.5153. Before conducting any measures, all adolescents provided written assent or consent while parents or legal guardians of adolescents under 18 years provided written consent for participation in the study.

To calculate the minimum sample size, we used a specific formula for cross-sectional studies contained in the EpiInfo software, version 7.2.2.16 (Georgia, United States). We set the population size at 471, which was the total number of students enrolled in the Institute high school grades at the end of 2017. The prevalence of mental disorders was established at 30% considering its prevalence in Brazilian adolescents [17]. We adopted an acceptable error of 5%, a confidence level of 95%, and a design effect of 1.1. From these settings, we found a minimum sample size of 211 adolescents. We increased the sample size by 10% (21 adolescents) to recover possible losses, making up a total sample size of 232 adolescents. The sample was obtained through simple random sampling. Participants were numerically representative of the grade and sex of the students attending the Institute. In cases where the adolescent declined participation in the study, the draw was disregarded, and a new drawing was carried out to replace them with another adolescent of the same age and sex. Details on data collection and inclusion/exclusion criteria have been published elsewhere [18].

### Movement behaviors: Physical activity and sedentary behavior

The movement behaviors of sleep, SB, LPA, and MVPA were measured and analyzed by the GT3X accelerometer (ActiGraph Corp, Pensacola, FL, USA) and ActiLife software (version 6.13.4, ActiGraph Corp, Pensacola, FL, USA), respectively. Adolescents wore the monitors on their right hip on an elastic belt for 8 consecutive days, including during sleep at night. Adolescents were instructed not to change their daily routine and that the accelerometer should be removed only for water-based activities, such as bathing and swimming. Participants were contacted daily through a mobile messaging app to ensure that the device was being appropriately used.

The accelerometer was initialized to collect data at a 30 Hz sampling rate and used the normal filter. The data were reintegrated into 15-s epochs. Non-wear time was defined as consecutive zero counts/min that lasted for at least 20-min. To be included in the analysis, participants were required to reach a minimum of 10 h.day^-1^ of “wear time” (without considering sleep) for at least 6 days, including at least 1 weekend day. Participants were also required to wear the accelerometer for, at least, 3 nights (at least one weekend night).

To calculate sleep time, we evaluated daily graphs, inclinometer data, and converted these data into a Microsoft Excel comma-separated values file. Together, these data helped us to detect the moment when the teenager woke up in the morning and went to sleep at night. These bed/wake times were used to create subject log diaries and to estimate sleep per night. The cut-points proposed by Romanzini et al. [19], validated for Brazilian adolescents were used to classify MVPA, LPA, and SB. More details regarding accelerometer setting and usage are available elsewhere [18].

### Mental health indicator

To assess depression/anxiety, we used the Portuguese version of the 12-item General Health Questionnaire (GHQ-12) [20], validated for application in Brazilian adolescents [21]. It is a self-report instrument based on the last few weeks, suitable for screening depression/anxiety and social dysfunction [22–25]. Each question has four possible answers to describe the presence and intensity of the mental disorder: “not at all”, “no more than usual”, “somewhat more than usual”, and “much more than usual”. To quantify the scale, we applied the “GHQ method” (0-0-1-1) [26], with the total scoring system ranging from 0 to 12. Thus, the first two answers describe a normal mental state and were coded as "0". The last two responses indicate an altered mental status and were coded as "1". We calculated the score for each adolescent, with higher scores indicating a worse mental health status.

To assess the GHQ-12 reliability, we used the Cronbach’s α, a measure of internal consistency. A coefficient higher than 0.7 indicated good reliability [27]. We used factor analysis to estimate a mental health scores from the GHQ-12. The Kaiser-Meyer-Olkin (KMO) test measured the adequacy of the dataset to perform factor analysis. A value higher than 0.7 indicated the suitability of the dataset [28]. Oblique rotation was applied to extract the factor structure. We used eigenvalue > 1.0 and examination of the scree test to determine the number of factors to be retained. Factor analysis was conducted in R with the *psych* package.

### Covariates

Adolescents reported sociodemographic characteristics of sex, age, and socioeconomic status (SES). SES was measured through a specific questionnaire proposed by the Brazilian Association of Survey Companies [29]. The questionnaire assigns different scores based on residence characteristics and the educational level of the household head. The score ranges from 0 to 100 points, with higher scores representing a better financial status.

Adolescents’ weight (kg) and height (cm) were measured by a digital scale (Plenna^®^, São Paulo, Brazil) and a portable stadiometer (Sanny Medical^®^, São Paulo, Brazil), according to Lohman et al. [30]. Adolescents were barefoot, wore light clothes, and removed any ornaments. BMI was calculated through the formula (weight (kg)/height (m)^2^), and classified by z-score, according to sex and age [31].

### Statistical analysis

The analyses were performed in the R Statistical Software system (version 3.6.3) and the alpha level was set at 0.05. Descriptive statistics (medians and percentiles) were calculated to describe the participants’ characteristics.

Compositional descriptive statistics, including compositional geometric means (central tendency), variation matrix (dispersion), and geometric mean bar plots (relative to behavioral profiles for mental health score), were calculated [7]. For the variation matrix, values closer to zero indicate higher co-dependence between the two behaviors included in the analyses, while values close to 1 indicate that two behaviors are least co-dependent [7].

Compositional analysis was conducted in the R packages *compositions* and *robcompositions*. Average minutes per day of sleep, SB, LPA, and MVPA across valid days were calculated. After that, the proportion of the 24 hours spent in these behaviors were normalized for each participant to collectively sum to 1440 min (24-h) [5]. Compositional descriptive statistics of geometric means and variation matrix were calculated for the movement behaviors. The geometric mean is a method to assess the central tendency of the composition after the movement behaviors have been normalized. The variation matrix describes the dispersion and was derived by calculating the variation of the logarithms of all possible pair-wise ratios (e.g., the variation of *ln*(SB/LPA)). Lower values (close to zero) indicate that the time spent between the two movement behaviors were highly co-dependent [5].

Participants’ compositional movement behaviors were expressed as three ilr coordinates including (1) sleep: SB; (2) LPA: the geometric mean of sleep and SB; and (3) MVPA: the geometric mean of sleep, SB, and LPA [32]. These ilr coordinates were used as exposure variables in multiple linear regression models to verify their association with GHQ-12 factor scores (outcomes). Analyses included the covariates sex, age, and SES. This approach enables all the four movement behaviors (sleep, SB, LPA, and MVPA) to act as the independent variable while considering the relative time spent in the other behaviors [8]. The significance of the composition was evaluated using Chi-square type II analysis of deviance tests of the regressions. Compositional isotemporal substitution analysis considered the coefficients from the regression model [5] to estimate the expected change in the GHQ-12 factors’ scores due to the reallocation of 10, 30, and 60 min for all possible combinations between sleep, SB, LPA, and MVPA.

## Results

In total, we invited 247 adolescents to participate in this study. Of these, 228 adolescents turned in the signed consent papers and completed the survey, but 11 were removed from the sample for not using the accelerometer appropriately. When comparing the characteristics between adolescents removed from the sample (n = 11) and the ones included in the study (N = 217), there was no significant difference (p>0,05) for most of the compared parameters (weight, height, age, sex, and prevalence of depression/anxiety). Differences were observed between these two groups in the variables of BMI and SES classes. Therefore, the final sample comprised 217 adolescents, of which 49.3% were female.

The majority of the adolescents (80.65%) wore the accelerometer for 7 days, while the remaining wore it for 6 days. Regarding the sleeping measure, 45.6% (n = 99) wore the device for 6 nights, 12.9% (n = 28) wore it for 7 nights, while 25.3% (n = 55) wore it for 5 nights. A small number of participants wore the accelerometer for 3 (3.2%; n = 7) and 4 (12.9%; n = 28) nights. Mean accelerometer usage time was 1376 min (22 hours and 56 minutes). Table 1 shows the sample’s characteristics.

**Table 1:** Descriptive statistics.

Variables	Male (*n* = 110)	Female (*n* = 107)	Total (N = 217)
**Age** (years)	16.0 (15.0–17.0)	16.0 (15.0–17.0)	16.0 (15.0–17.0)
**BMI** (kg/m^2^)	21.4 (19.4–24.9)	21.0 (19.0–23.5)	21.2 (19.2–24.5)
**SES** (points)	32.0 (27.8–39.3)	31.0 (24.0–35.0)	32.0 (26.0–37.0)

BMI, body mass index; SES, socioeconomic status. Data are presented as median (25^th^– 75^th^ percentile)

### Factor analysis

The GHQ-12 Cronbach’s α was 0.87, indicating a good internal consistency. The KMO value of 0.89 confirmed that the dataset supported the performance of the analysis. Results identified two factors, accounting for 62.1% of the total variance. Factor 1 was defined by seven questions related to depression/anxiety (questions 3, 4, 8, 9, 10, 11, and 12). Factor 2 included five questions related to social dysfunction (questions 1, 2, 5, 6, and 7). Factor loadings are presented in S1 File. Only Factor 1 (depression/anxiety score) was associated with the composition of movement behaviors and included in the regression models and used for compositional isotemporal substitutions.

### Compositional analysis

Geometric means of the movement behavior and corresponding % of the 24-h, after normalizing them to 1440 min/day, are shown in Table 2. In the full sample, the compositional means indicated that more than half of the day was spent in SB (55.4%), followed by sleep (30.3%), LPA (10.5%), and MPVA (3.7%). The contribution of the movement behavior over 24 hours is provided in S2 File.

**Table 2:** Geometric means for movement behaviors.

Movement Behavior	Min/day	% of 24-h
**Sleep**	436.4	30.3
**SB**	798.4	55.4
**LPA**	152.4	10.5
**MVPA**	52.8	3.7

SB, sedentary behavior; LPA, light physical activity; MVPA, moderate-to-vigorous physical activity. Movement behaviors were normalized to 1440 min.

Table 3 presents the pair-wise log-ratio variation matrix between each pair of movement behaviors. Sleep and SB presented the highest co-dependency (0.03), followed by sleep and LPA (0.08), and by SB and LPA (0.08), which imply in a high co-dependence between each pair of variables. The lowest levels of co-dependency were observed between MVPA and sleep (0.19), and MVPA and SB (0.18), which suggests that time spent in MVPA was the least co-dependent on the other behaviors.

**Table 3:** Pair-wise log-ratio variation matrix for the movement behaviors.

Movement Behavior	Sleep	SB	LPA	MVPA
**Sleep**	0.00	0.03	0.08	0.19
**SB**	0.03	0.00	0.08	0.18
**LPA**	0.08	0.08	0.00	0.09
**MVPA**	0.19	0.18	0.09	0.00

SB, sedentary behavior; LPA, light physical activity; MVPA, moderate-to-vigorous physical activity.

**Fig 1:**
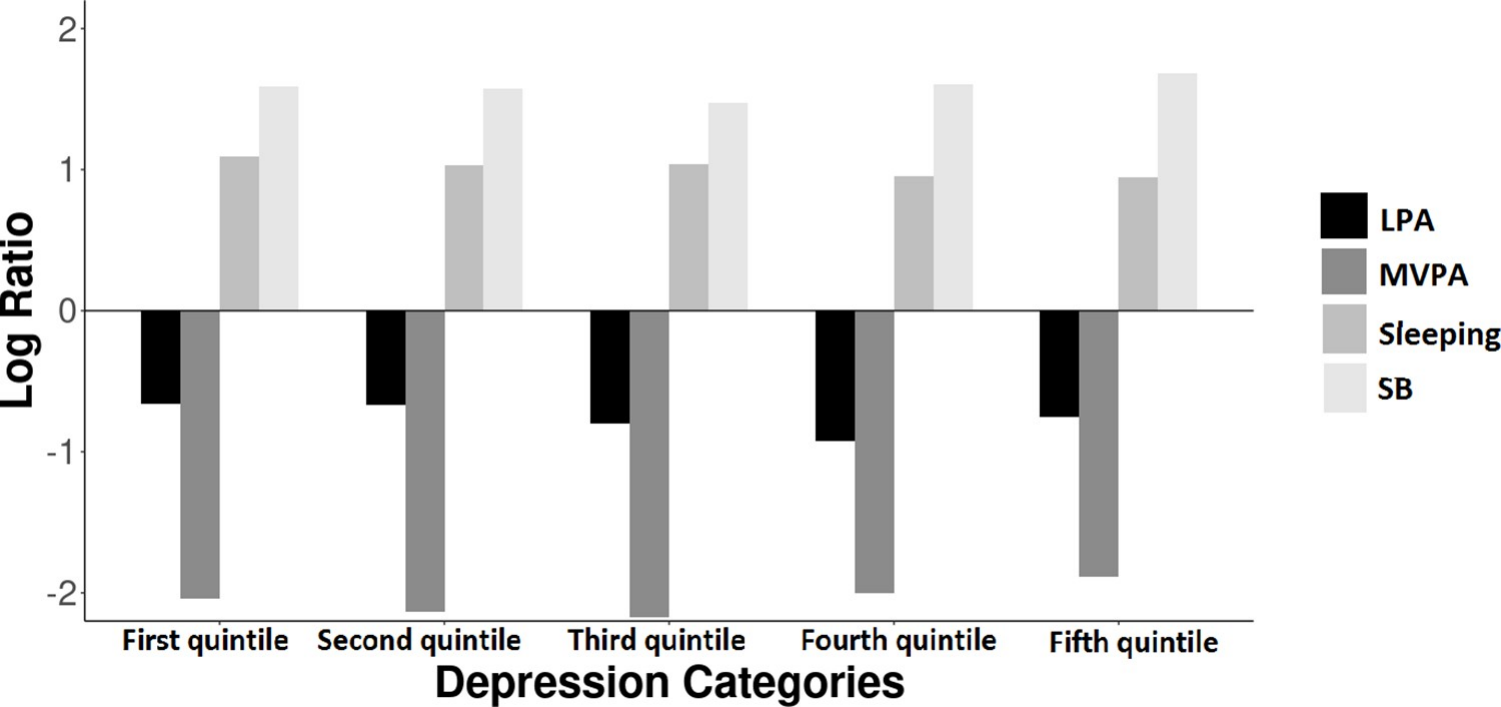
Compositional geometric mean bar plot for quintiles of depression/anxiety among adolescents. LPA, Light physical activity; MVPA, Moderate-to-vigorous physical activity; SB, Sedentary behavior.

Fig 1 presents the compositional geometric mean bar plots for quintiles of the depression/anxiety score. The proportion of time spent in LPA and MVPA was lower, while the proportion of time spent in SB and sleeping was higher in all quintiles relative to the entire sample. Adolescents from the highest quintile of the depression/anxiety score tended to have a slightly higher time spent in SB and sleeping and lower time in LPA and MVPA than those adolescents from the first quintile.

### Linear regression models

Chi-squared Type II analysis of deviance indicated that the movement behavior composition (*p* < 0.05) and sex (*p* < 0.001) were associated with the depression/anxiety score, but not with age or SES. In the multiple regression analysis, after adjusting for sex, age, and SES, the proportion of time spent in SB (0.925, *p* < 0.05) and LPA (-0.887, *p* < 0.05) was associated with the depression/anxiety score. The proportion of time spent in sleep and MVPA were not associated with the depression/anxiety score. The R2 value of all regressions was 0.149.

### Compositional isotemporal substitutions

Table 4 shows the significant results of the compositional isotemporal substitutions for reallocations of 10, 30, and 60 min between two specific movement behaviors. Reallocating 10, 30, and 60 min from SB to LPA reduced the depression/anxiety score by 0.06, 0.17, and 0.32, respectively. Besides that, reallocating 10, 30, and 60 min from LPA to SB increased the depression/anxiety score by 0.06, 0.20, and 0.44, respectively. Finally, replacing 10, 30, and 60 min of LPA with MVPA resulted in an increase of 0.12, 0.35, and 0.69 on the depression/anxiety score, respectively. All other possible alternatives for reallocating time between movement behavior were not significant.

**Table 4:** Predicted changes in the depression/anxiety score associated with the reallocation of 10, 30, and 60 min between movement behaviors.

**↓ 10 min**	**↑ 10 min**	**Depression/anxiety score (95% CI)**
SB	LPA	-0.06 (-0.10, -0.01)
LPA	SB	0.06 (0.01, 0.11)
LPA	MVPA	0.12 (0.008, 0.24)
**↓ 30 min**	**↑ 30 min**	
SB	LPA	-0.17 (-0.30, -0.03)
LPA	SB	0.20 (0.04, 0.36)
LPA	MVPA	0.35 (0.03, 0.67)
**↓ 60 min**	**↑ 60 min**	
SB	LPA	-0.32 (-0.57, -0.06)
LPA	SB	0.44 (0.08, 0.81)
LPA	MVPA	0.69 (0.06, 1.32)

SB, sedentary behavior; LPA, light physical activity; MVPA, moderate-to-vigorous physical activity. All analyses were adjusted for sex, age, and SES.

## Discussion

To our knowledge, this is the first study to carry out a compositional and isotemporal substitution data analysis approach to verify the association between movement behaviors within the 24 hours of the day and mental health in adolescents. The movement behavior composition was associated with the depression/anxiety score, which suggests the critical importance of the 24-h time distribution to mental health during adolescence. Collectively, our findings indicate the beneficial association between reallocating time away from SB to LPA and mental health. In fact, this result adds evidence and extends the emerging literature based on compositional analysis by showing that the substitution of a minimum amount of time, as little as 10 minutes of the day from SB to LPA is associated with improved mental health. These results can have a crucial implication in public health messages regarding depression/anxiety to encourage adolescents to start a behavioral shift by sitting less and moving more through light-intensity activities. Besides that, we reinforce the need for a holistic intervention approach, targeting the full range of possible movement behaviors in a day, instead of a single one (e.g., MVPA) [16, 18], to improve mental health in adolescents. Also, our results aligned with previous studies that identified the two factors structure of the GHQ-12, comprising the depression/anxiety and the social dysfunction factors [22–24, 33].

A growing body of evidence has suggested the detrimental effects of SB on depression/anxiety scores among adolescents [2, 3]. Our results extended the literature by showing that reallocating time spent in SB to LPA was beneficially associated with depression/anxiety in adolescents. By applying a statistical approach that considers the finite time of the day (24-h), the results highlighted that a change in a minimum amount of 10 minutes per day is associated with mental health improvement. It is noteworthy that better improvements in the score were seen with the replacement of longer proportions of time (30 and 60 minutes). These results suggested that every minute of the 24-h period matters when the focus is to improve mental health in adolescence. In addition, little is known about the association between LPA and depression/anxiety. The scarcity of these data must reflect the difficulty of measuring LPA, which can only be accurately assessed using an objective method (e.g., direct observation). These results have a practical relevance as they suggest that future interventions should focus on promoting LPA to replace SB. LPA should be understood as the gateway for the adolescent to be regularly involved in some type of activity that can positively affect their levels of depression/anxiety.

Another important finding related to the reallocation of time between SB and LPA is the asymmetry of the results. These asymmetric relationships are in line with previous studies involving compositional isotemporal substitution [5, 8, 11], and can only be observed by applying this statistical approach [8]. For example, reallocating 60 min from SB to LPA was associated with a reduction of 0.32 in the depression/anxiety score, while the inverse was associated with a greater change in this score (+0.44). These patterns of asymmetry were observed for all the substitutions between SB and LPA, independently of the amount of time involved. That is, the detrimental effects of a time reduction in LPA are greater than the estimated beneficial results that occur from its increase. A possible explanation for this fact relies on the distribution of time among the movement behaviors within the 24-h period [5]. Considering our sample, reallocating 60 minutes from LPA represents approximately 40% of this movement behavior while reallocating 60 minutes from SB only represents 7.5%. Therefore, any reallocation from LPA constitutes a substantial proportion of its time, which is not true for the SB. Together, these results reinforce the need for interventions that, at least, promote the maintenance of the LPA levels for reducing depression/anxiety scores.

Although most of the research has shown a beneficial association between MVPA and depression/anxiety levels [4, 34, 35], in the present study we found that replacing time from LPA to MVPA was associated with higher depression/anxiety scores. To better interpret this result, we believe that it is necessary to understand the context in which the study subjects live. The adolescents included in this study spend more than 9 hours (from 7:30 am to 4:50 pm) of their weekdays in school and take high school classes along with technical education. One of the technical courses included in the Institute curriculum is agriculture, which demands a high physical effort from the students. Besides that, part of our sample lives in and works on farms, where work can also be exhaustive. Considering that, we hypothesize that the relationship between MVPA and depression/anxiety scores may be moderated by the context in which this activity happens. In line with this, Dumuid et al. [11] evaluated the association between quality of life and movement behavior composition of 5,855 children aged 9–11 from countries with different human development indexes. The results showed that children from countries of higher human development index had higher associations between quality of life and MVPA than children from other countries [11]. To explain the results, it was speculated that the MVPA performed by children from countries of low development may be predominantly related to work, household chores, or active transport [11]. Together, these results suggested that the type of MVPA in which the adolescent is involved appears to play a critical role in different aspects of mental health.

Strengths of this study include the objective measurement of adolescents’ movement behaviors, the use of accelerometer cut points developed with Brazilian adolescents, and the compositional data analysis. This approach allowed us to consider the co-dependent relationships among the four possible movement behaviors within a finite 24-h day. Another strength that gives reliability to the results is the high adherence of the participants to the protocol, considering the non-wear time of 20 minutes and the fact that most of the adolescents wore the accelerometer 7 days for approximately 23 hours per day on average, probably due to the automated daily electronic reminders to wear the device. Nonetheless, the study has some limitations. Although our analyzes were theoretically guided, using a compositional approach and comparing subjects with similar age, sex and socioeconomic background in a multiple regression framework, the cross-sectional nature of the data precludes us from making more rigorous causal inferences. Additionally, the accelerometer was not used during water-based activities (e.g., swimming), which could have underestimated light and moderate-to-vigorous physical activities.

## Conclusions

In conclusion, the movement behavior distribution of the day was found to be associated with depression/anxiety scores among adolescents. The amount of time spent in SB relative to the other movement behaviors was detrimentally associated with the depression/anxiety scores, while time spent in LPA was beneficially associated with it, in adolescents. Replacing as little as 10 minutes of SB with LPA was associated with improvements in adolescents’ mental health. Finally, the detrimental association between the reallocation of time from LPA to MVPA and the depression/anxiety score may suggest that the type of MVPA is an important factor to be considered. We suggest that longitudinal and experimental studies may provide further insights into the associations. Finally, qualitative data should be investigated to better understand the determinants related to the 24-h movement behavior distribution among adolescents.

## Supporting information

S1 File Factor loading of the GHQ-12 questions. (DOCX)

S2 File Distribution of the 24-h period of the day among the four possible movement behaviors. SB, sedentary behavior; SD, sleep duration; LPA, light physical activity; MVPA, moderate-to-vigorous physical activity.
(DOCX)

## References

[1] Choi KW, Chen CY, Stein MB, Klimentidis YC, Wang MJ, Koenen KC, et al. Assessment of Bidirectional Relationships Between Physical Activity and Depression Among Adults: A 2-Sample Mendelian Randomization Study. *JAMA Psychiatry*. 2019; 76: 399–408. doi: 10.1001/jamapsychiatry.2018.4175 30673066 PMC6450288

[2] Bélair MA, Kohen DE, Kingsbury M, Colman I. Relationship between leisure time physical activity, sedentary behaviour and symptoms of depression and anxiety: evidence from a populationbased sample of Canadian adolescents. *BMJ Open*. 2018; 8: e021119. doi: 10.1136/bmjopen-2017-021119 30337306 PMC6196847

[3] Huang Y, Li L, Gan Y, Wang C, Jiang H, Cao S, et al. Sedentary behaviors and risk of depression: a meta-analysis of prospective studies. *Transl Psychiatry*. 2020; 10: 26. doi: 10.1038/s41398-020-0715-z 32066686 PMC7026102

[4] Oberste M, Medele M, Javelle F, Wunram HL, Walter D, Bloch W, et al. Physical Activity for the Treatment of Adolescent Depression: A Systematic Review and Meta-Analysis. *Front Physiol*. 2020; 11:185. doi: 10.3389/fphys.2020.00185 32265725 PMC7096373

[5] Chastin SF, Palarea-Albaladejo J, Dontje ML, Skelton DA. Combined Effects of Time Spent in Physical Activity, Sedentary Behaviors and Sleep on Obesity and Cardio-Metabolic Health Markers: A Novel Compositional Data Analysis Approach. *PLoS One*. 2015; 10: e0139984. 10.1371/journal.pone.0139984 26461112 PMC4604082

[6] Pedišić Z, Dumuid D, Olds TS. Integrating sleep, sedentary behaviour, and physical activity research in the emerging field of time-use epidemiology: definitions, concepts, statistical methods, theoretical framework, and future directions. *Kinesiology*. 2017; 49.

[7] Fairclough SJ, Dumuid D, Taylor S, Curry WB, McGrane B, Stratton G, et al. Fitness, fatness and the reallocation of time between children’s daily movement behaviours: an analysis of compositional data. *Int J Behav Nutr Phys Act*. 2017; 14. doi: 10.1186/s12966-017-0521-z 28486972 PMC5424384

[8] Biddle GJH, Edwardson CL, Henson J, Davies MJ, Khunti K, Roulands AV, et al. Associations of Physical Behaviours and Behavioural Reallocations with Markers of Metabolic Health: A Compositional Data Analysis. *Int J Environ Res Public Health*. 2018; 15: 2280. doi: 10.3390/ijerph15102280 30336601 PMC6210541

[9] Chaput JP, Saunders TJ, Carson V. Interactions between sleep, movement and other non-movement behaviours in the pathogenesis of childhood obesity. *Obes Rev*. 2017; 18: S7–14. doi: 10.1111/obr.12508 28164448

[10] Curtis RG, Dumuid D, Olds T, Plotnikoff R, Vandelonotte C, Ryan J, et al. The Association Between Time-Use Behaviors and Physical and Mental Well-Being in Adults: A Compositional Isotemporal Substitution Analysis. *J Phys Act Health*. 2020; 17: 197–203. doi: 10.1123/jpah.2018-0687 31918406

[11] Dumuid D, Maher C, Lewis LK, Stanford TE, Fernández JAM, Ratcliffe J, et al. Human development index, children’s health-related quality of life and movement behaviors: a compositional data analysis. *Qual Life Res*. 2018; 27: 1473–82. doi: 10.1007/s11136-018-1791-x 29362939 PMC7484943

[12] Aitchison J. The statistical analysis of compositional data. *J R Stat Soc Series B Stat Methodol*. 1982; 44: 139–77.

[13] Carson V, Tremblay MS, Chaput JP, McGregor D, Chastin S. Compositional analyses of the associations between sedentary time, different intensities of physical activity, and cardiometabolic biomarkers among children and youth from the United States. *Plos One*. 2019; 14: e0220009. doi: 10.1371/journal.pone.0220009 31329609 PMC6645531

[14] Carson V, Tremblay MS, Chaput JP, Chastin SF. Associations between sleep duration, sedentary time, physical activity, and health indicators among Canadian children and youth using compositional analyses. *Appl Physiol Nutr Metab*. 2016; 41: S294–302. doi: 10.1139/apnm-2016-0026 27306435

[15] Stefelova N, Dygryn J, Hron K, Gába A, Rubín L, Palarea-Albaladejo J. Robust Compositional Analysis of Physical Activity and Sedentary Behaviour Data. *Int J Environ Res Public Health*. 2018; 15: 2248. doi: 10.3390/ijerph15102248 30322203 PMC6210094

[16] Cruz BP, Alfonso-Rosa RM, McGregor D, Chastin SF, Palarea-Albaladejo J, Cruz JP. Sedentary behaviour is associated with depression symptoms: Compositional data analysis from a representative sample of 3233 US adults and older adults assessed with accelerometers. *J Affect Disord*. 2020; 265. 10.1016/j.jad.2020.01.023 31959584

[17] Lopes CS, Abreu GA, Santos DF, Menezes PR, Carvalho KMB, Cunha CF, et al. ERICA: prevalence of common mental disorders in Brazilian adolescents. *Rev Saúde Públ*. 2016; 50:S14. 10.1590/s01518-8787.2016050006690 PMC4767030 26910549

[18] Faria FR, Miranda VPN, Howe CA, Sasaki JE, Amorim PRS. Behavioral classes related to physical activity and sedentary behavior on the evaluation of health and mental outcomes among Brazilian adolescents. *PLoS One*. 2020; 15: e0234374. doi: 10.1371/journal.pone.0234374 32569320 PMC7307735

[19] Romanzini M, Petroski EL, Ohara D, Dourado AC, Reichert FF. Calibration of ActiGraph GT3X, Actical and RT3 accelerometers in adolescents. *Eur J Sport Sci*. 2014; 14: 91–9. doi: 10.1080/17461391.2012.732614 24533499

[20] Goldberg DP. The detection of psychiatric illness by questionnaire: a technique for the identification and assessment of non-psychotic psychiatric illness. London: University Press; 1972.

[21] Mari JJ, Williams P. A comparison of the validity of two psychiatric screening questionnaires (GHQ-12 and SRQ-20) in Brazil, using Relative Operating Characteristic (ROC) analysis. *Psychol Med*. 1985; 15: 651–9. 10.1017/s0033291700031500 4048323

[22] Allison KR, Adlaf EM, Irving HM, Hatch JL, Smith TF, Dwyer JJM, et al. Relationship of vigorous physical activity to psychologic distress among adolescents. *J Adolesc Health*. 2005; 37: 164–6. doi: 10.1016/j.jadohealth.2004.08.017 16026729

[23] Gouveia VV, Barbosa GA, Andrade EO, Carneiro MB. Factorial validity and reliability of the General Health Questionnaire (GHQ-12) in the Brazilian physician population. *Cad Saude Publica*. 2010; 26: 1439–45. 10.1590/S0102-311X2010000700023 20694370

[24] Schmitz N, Kruse J, Tress W. Psychometric properties of the General Health Questionnaire (GHQ-12) in a German primary care sample. *Acta Psychiatr Scand*. 1999; 100: 462–68. doi: 10.1111/j.1600-0447.1999.tb10898.x 10626926

[25] Namjoo S, Shaghaghi A, Sarbaksh P, Allahverdipour H, Pakpour AH. Psychometric properties of the General Health Questionnaire (GHQ-12) to be applied for the Iranian elder population. *Aging Ment Health*. 2017; 21: 1047–51. doi: 10.1080/13607863.2016.1196337 27333198

[26] Goldberg DP, Gater R, Sartorius N, Ustun TB, Piccinelli M, Gureje O, et al. The validity of two versions of the GHQ in the WHO study of mental illness in general health care. *Psychol Med*. 1997; 27. doi: 10.1017/s0033291796004242 9122299

[27] Fleiss JL. Design and analysis of clinical experiments. New York: John Wiley & Sons; 1986.

[28] Kaiser HF. An index of factor simplicity. *Psychometrika*. 1974; 39: 31–6.

[29] Brazilian Association of Survey Companies. 2016. http://www.abep.org/criterio-brasil (accessed on September 10, 2017).

[30] Lohman TG, Roche AF, Martorell R. Anthropometric Standardization Reference Manual. Champaign: Human Kinects; 1988.

[31] de Onis M, Onyango AW, Borghi E, Siyam A, Nishida C, Siekmann J. Development of a WHO growth reference for school-aged children and adolescents. *Bull World Health Organ*. 2007; 85: 660–7. doi: 10.2471/blt.07.043497 18026621 PMC2636412

[32] Foley L, Dumuid D, Atkin AJ, Olds T, Ogilvie D. Patterns of health behaviour associated with active travel: a compositional data analysis. *Int J Behav Nutr Phys Act*. 2018; 15. 10.1186/s12966-018-0662-8 PMC5861598 29562923

[33] Wong KC, O’Driscoll MP. Psychometric properties of the General Health Questionnaire-12 in a sample of Hong Kong employees. *Psychol Health Med*. 2016; 21: 975–80. doi: 10.1080/13548506.2016.1140901 26806584

[34] Kandola A, Ashdown-Franks G, Hendrikse J, Sabiston CM, Stubbs B. Physical activity and depression: Towards understanding the antidepressant mechanisms of physical activity. *Neurosci Biobehav Rev*. 2019; 107: 525–39. doi: 10.1016/j.neubiorev.2019.09.040 31586447

[35] Nakagawa T, Koan I, Chen C, Matsubara T, Hagiwara K, Lei H, et al. Regular Moderate- to Vigorous-Intensity Physical Activity Rather Than Walking Is Associated with Enhanced Cognitive Functions and Mental Health in Young Adults. *Int J Environ Res Public Health*. 2020; 17. doi: 10.3390/ijerph17020614 PMC7014044 31963639

