# Supplementary material for: Time-use movement behaviors are associated with scores of depression/anxiety among adolescents: A compositional data analysis
Source: PLoS One. 2022 Dec 30;17(12):e0279401. doi: 10.1371/journal.pone.0279401 (PMC9803290; doi:10.1371/journal.pone.0279401)
Supplement: S1 File — (DOCX) [file pone.0279401.s001.docx]

**Supplementary Material S1. Factor loading of the GHQ-12 questions.**

| **Variables** | **Factor 1 (Depression/anxiety)** | **Factor 2 (Social Dysfunction)** |
| --- | --- | --- |
| **1. Able to concentrate** |  | 0.6 |
| **2. Lost sleep over worry** |  | 0.8 |
| **3. Play a useful part in things** | 0.9 |  |
| **4. Capable of making decisions** | 0.5 |  |
| **5. Constantly under strain** |  | 0.7 |
| **6. Could not overcome difficulties** |  | 0.5 |
| **7. Enjoy day-to-day activities** |  | 0.6 |
| **8. Face up to problems** | 0.6 |  |
| **9. Feeling unhappy and depressed** | 0.7 |  |
| **10. Loss of self-confidence** | 0.7 |  |
| **11. Thinking of self as worthless** | 0.8 |  |
| **12. Feeling reasonably happy** | 0.6 |  |
