# Supplementary material for: Time-use movement behaviors are associated with scores of depression/anxiety among adolescents: A compositional data analysis
Source: PLoS One. 2022 Dec 30;17(12):e0279401. doi: 10.1371/journal.pone.0279401 (PMC9803290; doi:10.1371/journal.pone.0279401)
Supplement: S2 File — SB, sedentary behavior; SD, sleep duration; LPA, light physical activity; MVPA, moderate-to-vigorous physical activity. (DOCX) [file pone.0279401.s002.docx]

**Supplementary Material S2. Distribution of the 24-h period of the day among the four possible movement behaviors.**

SB, sedentary behavior; SD, sleep duration; LPA, light physical activity; MVPA, moderate-to-vigorous physical activity.
